# Precision Diagnostics in Prostate Cancer: Integrating Biomarkers, Imaging, Genomics, and Artificial Intelligence in Contemporary United States Practice

**DOI:** 10.3390/medsci14050541

**Published:** 2026-09-02

**Authors:** Moustafa Kardjadj

**Affiliations:** dicentra, Toronto, ON M4W 3E2, Canada; drkardjadj@live.fr

**Keywords:** prostate cancer, precision diagnostics, prostate-specific antigen, biomarkers, multiparametric MRI, PI-RADS, prostate biopsy, PSMA PET/CT, genomic classifiers, artificial intelligence, digital pathology, micro-ultrasound, liquid biopsy, molecular imaging

## Abstract

Prostate cancer is the most commonly diagnosed non-cutaneous malignancy among men in the United States and remains a leading cause of cancer-related mortality. Its marked biological, molecular, and histopathological heterogeneity creates a central diagnostic challenge: identifying clinically significant disease while limiting unnecessary biopsy and overdiagnosis of tumors unlikely to affect survival or quality of life. Although prostate-specific antigen (PSA) remains the foundation of early detection, its limited cancer specificity has driven the development of increasingly risk-adapted diagnostic pathways. Contemporary evaluation integrates clinical risk assessment and PSA-derived measures with selectively used blood- and urine-based biomarkers, multiparametric magnetic resonance imaging (mpMRI), image-guided biopsy, histopathological classification, genomic risk assessment, and molecular imaging. Biomarkers such as the Prostate Health Index, 4Kscore, IsoPSA, MiCheck, SelectMDx, and ExoDx may refine biopsy decisions in appropriately selected patients but should be interpreted according to the clinical setting, decision threshold, and surrounding diagnostic pathway. Prostate MRI and PI-RADS-based assessment have become central to pre-biopsy evaluation, while MRI-targeted biopsy improves detection of Grade Group ≥ 2 disease. Increasing use of the transperineal biopsy route offers comparable cancer detection with a lower infectious risk. Following diagnosis, Grade Group, adverse histological features, clinical risk models, and selected tissue-based genomic classifiers provide complementary prognostic information. PSMA PET/CT has further improved staging of selected patients with higher-risk disease and localization of biochemical recurrence. Precision diagnostics must also account for disease phenotypes that may not be adequately represented by conventional PSA- and imaging-based pathways, including intraductal carcinoma, cribriform architecture, ductal adenocarcinoma, and neuroendocrine prostate cancer. Emerging approaches, including artificial intelligence-assisted MRI interpretation, digital pathology, high-frequency micro-ultrasound, liquid biopsy, alternative molecular radiotracers, and multi-omic integration, show increasing potential but remain at different stages of validation and clinical adoption. This review critically examines contemporary prostate cancer diagnostics within United States clinical practice, distinguishing established guideline-supported approaches from selectively used adjuncts and emerging technologies. Particular emphasis is placed on diagnostic performance in context, clinical utility, external validation, healthcare equity, regulatory considerations, and the need to demonstrate that increasing diagnostic complexity translates into meaningful improvements in patient care.

## 1. Introduction

Prostate cancer is the most commonly diagnosed non-cutaneous malignancy among men in the United States and remains the second leading cause of cancer-related death in this population [1,2]. The American Cancer Society estimates that approximately 333,830 new cases and 36,320 deaths will occur in the United States in 2026 [1]. Prostate cancer is biologically and clinically heterogeneous, ranging from indolent tumors that may never become clinically consequential to aggressive disease associated with metastatic progression and cancer-specific mortality [3]. This heterogeneity represents a central challenge in prostate cancer diagnostics: identifying clinically significant disease while avoiding unnecessary investigation and treatment of tumors unlikely to affect survival or quality of life.

The introduction of prostate-specific antigen (PSA) testing transformed prostate cancer detection by increasing the diagnosis of localized disease and contributing to reductions in prostate cancer mortality [4]. However, PSA is prostate-specific rather than cancer-specific, and widespread PSA-based screening has also been associated with false-positive evaluations, unnecessary biopsies, overdiagnosis, and detection of low-risk tumors [4,5]. These concerns contributed to the 2012 recommendation by the United States Preventive Services Task Force (USPSTF) against routine PSA-based screening. In 2018, the USPSTF subsequently recommended individualized decision-making for men aged 55–69 years after discussion of the potential benefits and harms of screening [5].

Contemporary screening strategies increasingly emphasize shared decision-making and individualized risk assessment. The American Urological Association/Society of Urologic Oncology (AUA/SUO), National Comprehensive Cancer Network (NCCN), and European Association of Urology (EAU) recognize that screening decisions should consider age, life expectancy, prior PSA measurements, family history, ancestry, germline susceptibility, and patient preferences [6,7,8]. The AUA/SUO recommends offering screening beginning at 40–45 years of age to individuals at increased risk, including those with Black ancestry, germline mutations associated with prostate cancer susceptibility, or a strong family history [6]. Pathogenic variants involving genes such as BRCA2, BRCA1, and HOXB13 may further inform individualized risk assessment and screening discussions [6,8].

Despite its central role in early detection, PSA alone cannot reliably distinguish clinically significant prostate cancer from benign prostatic conditions. Elevated PSA concentrations may occur in benign prostatic hyperplasia, prostatitis, urinary tract infection, urinary retention, and following prostatic manipulation [6,9]. Contemporary diagnostic pathways therefore combine PSA and PSA-derived parameters with clinical risk assessment and, when appropriate, secondary blood- or urine-based biomarkers, multiparametric magnetic resonance imaging (mpMRI), and image-guided biopsy [7,8,9]. Histopathological confirmation remains fundamental, while selected clinical nomograms and tissue-based molecular or genomic classifiers may provide additional prognostic information following diagnosis.

The diagnostic pathway is further influenced by tumor morphology and biological subtype. Although conventional acinar adenocarcinoma accounts for most prostate cancers, histological variants and architectural patterns may present distinct diagnostic challenges. The 2022 World Health Organization classification recognizes important refinements in prostate tumor classification, including entities associated with neuroendocrine differentiation and other clinically relevant pathological patterns [10]. Intraductal carcinoma of the prostate, cribriform architecture, and ductal adenocarcinoma are associated with distinct pathological and molecular characteristics that may influence diagnostic interpretation and risk assessment [11]. Neuroendocrine prostate cancer represents another diagnostically challenging phenotype because its clinical, pathological, biomarker, and imaging characteristics can differ substantially from conventional prostate adenocarcinoma [12].

Molecular imaging has also expanded the diagnostic evaluation of selected patients. Prostate-specific membrane antigen positron emission tomography/computed tomography (PSMA PET/CT) provides greater accuracy than conventional imaging for staging selected patients with high-risk disease, as demonstrated in the prospective proPSMA trial [13]. PSMA PET/CT also has an established role in evaluating biochemical recurrence, although its detection performance varies with PSA concentration, disease burden, tracer, and prior treatment [14]. These considerations are particularly relevant in diagnostically difficult or borderline settings in which conventional anatomical imaging may be equivocal.

Emerging technologies may further extend the precision diagnostic pathway but should be distinguished clearly from established or guideline-supported practice. Artificial intelligence-assisted prostate MRI has shown promising diagnostic performance in large multicenter validation studies, including the PI-CAI study, but remains an adjunctive technology rather than a universal standard-of-care approach [15]. Similarly, high-frequency micro-ultrasound has gained stronger prospective evidence following the OPTIMUM randomized clinical trial, which demonstrated noninferiority to MRI-guided biopsy for detection of clinically significant prostate cancer in biopsy-naïve men [16]. Digital pathology, liquid biopsy, novel molecular radiotracers, and multi-omic approaches are also under active investigation, but their clinical maturity and level of implementation vary substantially.

Accordingly, this review examines contemporary prostate cancer diagnostics within the United States clinical pathway, with particular emphasis on distinguishing established standard-of-care approaches, selectively used adjunctive tests, and emerging technologies. The review addresses early detection and PSA-based risk assessment, secondary biomarkers, mpMRI, image-guided biopsy, histopathology, genomic risk assessment, molecular staging and recurrence evaluation, histological variants and diagnostically challenging presentations, and emerging technologies including artificial intelligence, digital pathology, liquid biopsy, micro-ultrasound, advanced molecular imaging, and multi-omic integration. The focus is specifically on diagnostic, prognostic, and disease-characterization tools rather than on providing a comprehensive review of prostate cancer therapy.

### Literature Search Methodology

This article was conducted as a targeted narrative review rather than a formal systematic review or meta-analysis. PubMed/MEDLINE and Scopus were used as the principal bibliographic databases to identify peer-reviewed publications addressing contemporary prostate cancer diagnostic strategies. Google Scholar was used as a supplementary source for citation tracking and identification of additional relevant publications. The primary search period extended from January 2020 through August 2026. Earlier landmark studies were included when they established diagnostic concepts, validated widely used tests, or provided evidence that remains relevant to contemporary clinical practice.

The search strategy combined controlled vocabulary, including Medical Subject Headings (MeSH) where applicable, with free-text terms covering the major diagnostic domains evaluated in this review. Search concepts included “prostate cancer,” “prostatic neoplasms,” “prostate-specific antigen,” “PSA density,” “free PSA,” “prostate biomarkers,” “Prostate Health Index,” “4Kscore,” “IsoPSA,” “MiCheck,” “SelectMDx,” “ExoDx,” “ConfirmMDx,” “multiparametric MRI,” “PI-RADS,” “prostate biopsy,” “MRI-targeted biopsy,” “transperineal biopsy,” “micro-ultrasound,” “PRI-MUS,” “histopathology,” “intraductal carcinoma,” “cribriform,” “ductal adenocarcinoma,” “neuroendocrine prostate cancer,” “genomic classifier,” “Decipher,” “Oncotype DX,” “Prolaris,” “PSMA PET,” “biochemical recurrence,” “liquid biopsy,” “circulating tumor DNA,” “AR-V7,” “artificial intelligence,” “machine learning,” “digital pathology,” “FAPI PET,” and “multi-omics.” Terms within individual diagnostic domains were combined using OR, while domain-specific concepts were combined with prostate cancer terms using AND.

Eligible sources included clinical practice guidelines and consensus documents, randomized and prospective clinical studies, multicenter diagnostic-validation studies, large observational cohorts, systematic reviews, meta-analyses, and methodologically relevant diagnostic studies involving human prostate cancer populations. Priority was given to evidence applicable to contemporary United States practice. Publications evaluating emerging technologies were included when they provided clinically relevant validation, comparative diagnostic performance, regulatory information, or evidence concerning implementation and generalizability. Landmark studies published before 2020 were retained when necessary to describe the development or validation of established diagnostic modalities.

Studies were excluded when they were unrelated to prostate cancer detection, diagnosis, risk stratification, staging, or disease monitoring; were exclusively preclinical without a direct clinical diagnostic application; focused primarily on therapeutic efficacy without a relevant diagnostic or predictive biomarker component; or provided insufficient methodological information to evaluate the diagnostic claim being discussed. Case reports and small proof-of-concept studies were not prioritized when higher-level or externally validated evidence was available.

Titles and abstracts were assessed for relevance to the predefined diagnostic domains, followed by full-text review of potentially relevant publications. When multiple publications supported the same clinical statement, preference was given to current clinical practice guidelines, authoritative regulatory sources where regulatory status was discussed, randomized studies, systematic reviews and meta-analyses, and prospective multicenter validation cohorts. Reference lists of major guidelines, systematic reviews, and selected primary studies were also examined to identify additional relevant publications.

Evidence was interpreted according to study design, patient population, diagnostic threshold, reference standard, endpoint, external validation, and applicability to contemporary United States practice. Diagnostic performance measures such as sensitivity, specificity, negative predictive value, and area under the receiver-operating-characteristic curve were interpreted in relation to the population, cutoff, disease definition, and clinical setting in which they were reported rather than as universal properties of a diagnostic test. Established guideline-supported modalities were distinguished from selectively used adjunctive tests and emerging or investigational technologies. Because this was a narrative evidence synthesis rather than a formal systematic review, no pooled meta-analysis or formal quantitative risk-of-bias assessment was performed.

## 2. Biological Heterogeneity and Molecular Subtypes of Primary Disease

Prostate cancer is characterized by substantial intertumoral and intratumoral heterogeneity at the genomic, molecular, and histopathological levels. This heterogeneity contributes to the broad spectrum of clinical behavior observed in practice, ranging from indolent localized tumors to biologically aggressive cancers associated with early progression and metastatic dissemination [3]. Molecular profiling has demonstrated that prostate cancer is not a single biological entity but comprises distinct genomic subgroups that differ in oncogenic drivers, tumor-suppressor alterations, DNA-repair defects, and patterns of disease evolution [3].

### 2.1. Molecular Architecture of Prostate Adenocarcinoma

The Cancer Genome Atlas (TCGA) analysis established a molecular framework for primary prostate adenocarcinoma in which approximately three-quarters of tumors could be classified into seven major molecular subtypes defined by ERG, ETV1, ETV4, or FLI1 gene fusions or by recurrent SPOP, FOXA1, or IDH1 mutations [3]. Among these, rearrangements involving ERG, most commonly the androgen-regulated TMPRSS2–ERG fusion, represent the largest subgroup and occur in approximately 40–50% of primary prostate cancers in predominantly European-ancestry cohorts [3]. In contrast, SPOP mutations generally define an ETS-fusion-negative molecular subgroup and are frequently associated with additional genomic changes, including alterations involving CHD1 and androgen-receptor signaling pathways [3].

Alteration of the PTEN–PI3K–AKT pathway represents another important molecular event in prostate cancer. PTEN loss may occur through deletion, mutation, or other mechanisms of functional inactivation and is associated with increased PI3K/AKT pathway activity [3]. PTEN alterations are heterogeneous within primary tumors and become increasingly represented in biologically aggressive and advanced disease. Comparative genomic studies of metastatic prostate cancer have also demonstrated enrichment of alterations involving PTEN, TP53, RB1, androgen-receptor signaling, and other pathways during disease progression, illustrating the molecular evolution that can occur between primary and advanced disease [17]. These findings are important diagnostically because molecular profiles obtained from primary tissue may not fully represent the genomic landscape present later in the disease course.

Alterations in DNA damage-repair pathways constitute another clinically important component of prostate cancer heterogeneity. Pathogenic germline or somatic alterations may involve homologous recombination-repair genes such as BRCA2, BRCA1, ATM, CHEK2, PALB2, and related genes. Germline DNA-repair mutations are enriched in metastatic prostate cancer compared with localized disease, with BRCA2 representing one of the most frequently identified pathogenic germline alterations [18]. The diagnostic relevance of these findings extends beyond therapeutic selection: identification of a pathogenic germline alteration may refine hereditary cancer-risk assessment, prompt genetic counseling and cascade testing of relatives, and contribute to characterization of biologically aggressive disease. Somatic molecular profiling may additionally identify genomic features relevant to prognosis and subsequent disease management.

Importantly, comprehensive molecular sequencing is not required for every patient with newly diagnosed localized prostate cancer. Molecular information should complement rather than replace conventional clinicopathological assessment, including tumor grade, stage, PSA characteristics, imaging findings, family history, and histologic features. The clinical significance of an individual molecular alteration also depends on disease setting; alterations that are highly relevant in advanced disease should not automatically be interpreted as validated diagnostic or prognostic markers in otherwise low-risk localized cancer.

### 2.2. Histologic Heterogeneity and Diagnostic Implications

Although conventional acinar adenocarcinoma accounts for the large majority of prostate cancers, histologic heterogeneity has important diagnostic and prognostic implications. The fifth edition of the World Health Organization classification refined the terminology and diagnostic criteria for several prostatic tumor types and patterns, including intraductal carcinoma, ductal adenocarcinoma, neuroendocrine tumors, and unusual subtypes or patterns of acinar adenocarcinoma [10]. Recognition of these entities is particularly relevant to precision diagnostics because morphology may provide information that is not captured by PSA concentration, Grade Group, or conventional imaging alone.

Intraductal carcinoma of the prostate (IDC-P) is characterized by malignant epithelial proliferation within pre-existing prostatic ducts and acini with at least partial preservation of the basal-cell layer. In most cases occurring with invasive prostate cancer, IDC-P is considered to represent intraductal spread of biologically aggressive carcinoma rather than an isolated precursor lesion. IDC-P is associated with adverse pathological features and enrichment of genomic abnormalities, including alterations involving PTEN, TP53, and homologous recombination-repair pathways [11]. Its recognition on biopsy is therefore clinically important, but distinction from high-grade prostatic intraepithelial neoplasia, atypical intraductal proliferation, and invasive cribriform carcinoma can be challenging.

Recent joint recommendations from the Genitourinary Pathology Society and International Society of Urological Pathology provide a more standardized framework for the diagnosis and reporting of IDC-P and atypical intraductal proliferation [19]. In diagnostically equivocal cases, basal-cell immunohistochemistry may assist in distinguishing intraductal from invasive carcinoma. The updated consensus also emphasizes that atypical intraductal proliferation should be reserved for lesions suspicious for IDC-P that do not fully satisfy diagnostic criteria [19]. These refinements are relevant to clinical practice because inconsistent classification of intraductal lesions can affect grading, risk assessment, and subsequent diagnostic evaluation.

Cribriform growth is another clinically important architectural pattern. Invasive cribriform carcinoma consists of confluent malignant epithelial proliferations containing multiple glandular lumina and is associated with adverse pathological and clinical outcomes. Because considerable interobserver variability historically existed in recognizing cribriform morphology, the International Society of Urological Pathology developed a consensus definition to improve diagnostic reproducibility and standardized reporting [20]. The presence of invasive cribriform architecture or IDC-P is particularly relevant in tumors that might otherwise appear favorable on the basis of limited biopsy sampling or conventional Grade Group assessment.

Ductal adenocarcinoma represents a less common glandular form of prostate cancer with distinctive morphology and generally more aggressive clinicopathological behavior than conventional acinar adenocarcinoma [10,11]. Ductal and mixed ductal–acinar tumors may also present diagnostic challenges because PSA elevation may be less pronounced relative to tumor burden in some patients, and disease may arise in periurethral or centrally located regions. Accurate histopathological recognition is therefore important when clinical, biochemical, imaging, and pathological findings appear discordant.

Neuroendocrine prostate cancer (NEPC) represents a biologically distinct and diagnostically challenging phenotype. De novo small-cell neuroendocrine carcinoma is uncommon, whereas treatment-related neuroendocrine prostate cancer can emerge through lineage plasticity during advanced disease [10,12]. Neuroendocrine tumors may demonstrate relatively low PSA production despite substantial tumor burden, reflecting reduced dependence on conventional androgen-receptor signaling. Histopathological assessment, supported where appropriate by neuroendocrine markers such as synaptophysin, chromogranin, and INSM1, is therefore important when the clinical phenotype is discordant with serum PSA [12]. Reduced or heterogeneous PSMA expression may also limit the sensitivity of PSMA-targeted imaging in some neuroendocrine or poorly differentiated tumors, creating a setting in which alternative imaging and pathological evaluation may be required.

The diagnostic significance of histological heterogeneity is therefore not limited to tumor classification. Recognition of IDC-P, cribriform architecture, ductal differentiation, and neuroendocrine morphology can identify situations in which conventional PSA-based or imaging-based assessment may incompletely reflect disease biology. Integrating morphology with clinical features, imaging, germline or somatic testing when indicated, and standardized pathological reporting provides a more complete framework for precision diagnosis and risk characterization.

## 3. Epidemiology and Clinical Presentation

### 3.1. Epidemiologic Trends and the Impact of Screening Practices

Prostate cancer remains a major public health burden in the United States. Approximately one in eight men will be diagnosed with prostate cancer during his lifetime, although individual risk varies substantially according to age, ancestry, family history, inherited susceptibility, and other factors [1,2]. Incidence rises markedly with age, and the majority of diagnoses occur in older adults. The widespread introduction of prostate-specific antigen (PSA) testing in the late 1980s and early 1990s substantially altered prostate cancer epidemiology by increasing detection of previously unrecognized localized and asymptomatic disease [4].

Population incidence patterns have subsequently reflected changes in screening practice. Concerns regarding overdiagnosis and overtreatment contributed to the 2012 United States Preventive Services Task Force (USPSTF) Grade D recommendation against routine PSA-based screening [5]. PSA testing and overall prostate cancer incidence declined following this change, particularly through reductions in diagnoses of localized and lower-risk disease. However, population-based analyses subsequently identified increases in the incidence of advanced and metastatic prostate cancer. An analysis of Surveillance, Epidemiology, and End Results (SEER) data from 2004 to 2018 demonstrated that the incidence of distant metastatic prostate cancer increased significantly beginning around 2010–2011, with rising rates observed in multiple age groups [21].

These trends should be interpreted cautiously because observational data cannot establish that reduced PSA screening alone caused the increase in metastatic presentation. Changes in population demographics, diagnostic practices, imaging, and referral patterns may also contribute [21]. Nevertheless, these observations support the current emphasis on individualized, risk-adapted early detection rather than either indiscriminate screening or complete screening avoidance [5,6,7,8].

### 3.2. Clinical Presentation: Screen-Detected Versus Symptomatic Disease

Localized prostate cancer is usually asymptomatic and is commonly identified through PSA-based risk assessment followed by targeted diagnostic evaluation [6,7,8,9]. Lower urinary tract symptoms are common in older men but are nonspecific and more frequently reflect benign prostatic enlargement or bladder dysfunction; they should therefore not be interpreted as a surrogate for prostate cancer risk.

Symptoms directly attributable to prostate cancer are more typical of locally advanced or metastatic disease and may include urinary obstruction, hematuria, bone pain, or neurological manifestations from vertebral involvement. Certain histologic phenotypes, particularly ductal and neuroendocrine prostate cancer, may show disease burden disproportionate to serum PSA, reinforcing the need to interpret clinical presentation together with imaging and pathology [10,11,12].

### 3.3. Healthcare Equity and Disparities in Prostate Cancer Diagnosis

Marked racial, socioeconomic, and geographic disparities persist across the prostate cancer diagnostic continuum in the United States. Black men experience substantially higher prostate cancer incidence and mortality than non-Hispanic White men and are more likely to be diagnosed at younger ages and, in some populations, with more advanced disease [2,22]. These disparities are not adequately explained by a single biological or social factor.

Current evidence supports a multifactorial model in which ancestry-associated genetic susceptibility and tumor biology interact with structural and social determinants of health, healthcare access, screening patterns, insurance status, socioeconomic circumstances, geography, and representation in clinical research [22]. Barriers may arise at multiple stages of the diagnostic pathway, including access to primary care and PSA testing, timely urologic referral, availability of mpMRI, advanced molecular imaging, biomarker testing, high-quality biopsy services, genomic testing, and subspecialty pathology review. As precision diagnostic technologies become increasingly incorporated into practice, unequal availability of these technologies could widen existing disparities if implementation is not accompanied by attention to access and affordability.

Biological differences among populations also warrant investigation, but race should not be interpreted as a simple biological surrogate. Comparative molecular analyses have demonstrated differences in the distribution of some prostate cancer genomic and transcriptomic characteristics between tumors from African American and European American men. In a multi-institutional analysis of 1152 radical prostatectomy specimens, tumors from European American men demonstrated higher ERG/ETS expression, whereas tumors from African American men showed differential expression of several inflammatory, immune-response, hypoxia, and oxidative-stress pathways [23]. These observations are consistent with earlier reports of a lower frequency of TMPRSS2–ERG/ERG-positive tumors among men of African ancestry, but the clinical significance of these differences remains incompletely defined.

Such molecular observations should therefore be interpreted alongside, rather than in place of, the substantial effects of access to care and structural inequity. Studies conducted in settings with more comparable access and treatment have demonstrated attenuation of some outcome disparities, underscoring that population-level differences cannot be attributed solely to inherent tumor biology [22].

Improving equity in precision prostate cancer diagnostics will require interventions across the diagnostic pathway. Priorities include improving access to risk-adapted early detection and timely diagnostic evaluation; expanding availability of high-quality mpMRI, biopsy, molecular imaging, and clinically indicated biomarker or genomic testing; increasing representation of historically underrepresented populations in diagnostic-validation studies; and ensuring that emerging technologies are validated in populations that reflect the diversity of United States clinical practice [22,23]. Equity should therefore be considered an integral component of diagnostic performance and clinical implementation rather than a separate downstream concern.

## 4. Stratified Diagnostic Strategies

No single test can reliably distinguish clinically significant prostate cancer from indolent disease across all clinical settings. Contemporary prostate cancer diagnosis therefore relies on a risk-adapted, sequential framework in which clinical variables, prostate-specific antigen (PSA), secondary biomarkers, magnetic resonance imaging (MRI), tissue sampling, histopathology, and selected molecular tests contribute different information at different stages of evaluation [6,7,8,9]. The principal diagnostic target is generally Grade Group (GG) ≥ 2 prostate cancer, although the clinical importance of an individual finding depends on age, life expectancy, comorbidity, baseline risk, tumor volume, histologic features, and patient preferences [9].

Importantly, the diagnostic pathway is not a fixed sequence in which every patient undergoes every available test. Additional investigations are most useful when their results have a realistic probability of changing the decision to proceed with biopsy, determining how biopsy should be performed, refining risk after tissue diagnosis, or identifying the location or biological characteristics of recurrent or metastatic disease [7,8,9]. This distinction is particularly relevant for secondary biomarkers and genomic classifiers, which are adjunctive rather than universally required tests.

Table 1 summarizes the major diagnostic modalities discussed in this review. Performance estimates are deliberately linked to the population, threshold, endpoint, and study from which they were derived rather than presented as universal properties of each test.

### 4.1. Initial Detection and Pre-Biopsy Risk Stratification

#### 4.1.1. PSA and PSA-Derived Risk Measures

PSA remains the primary laboratory marker used for prostate cancer early detection, but contemporary practice increasingly treats PSA as a continuous risk variable rather than a binary diagnostic test [6,7,8,9]. No PSA concentration perfectly separates benign disease, indolent cancer, and clinically significant cancer. Consequently, an isolated PSA elevation should be interpreted in relation to age, previous PSA values, prostate volume, family history, germline risk, medications, urinary symptoms, infection, recent instrumentation, and other clinical findings.

When a newly elevated PSA is identified, confirmation with repeat testing before proceeding to secondary biomarkers, imaging, or biopsy can reduce downstream evaluation of transient elevations [6]. PSA velocity should similarly not be used as the sole indication for secondary testing or biopsy because longitudinal change adds limited independent diagnostic information when absolute PSA and other clinical risk variables are already considered.

PSA density (PSAD), calculated as serum PSA divided by prostate volume, adds useful information because an identical PSA concentration may carry substantially different implications in a 30 mL versus an 80 mL prostate. PSAD is particularly useful when interpreted together with MRI. Although values around 0.15 ng/mL/cm^3^ have historically been used as decision points, PSAD is better regarded as a continuous risk variable rather than a universal cutoff. Contemporary risk-adapted approaches combine PI-RADS category and PSAD, recognizing that a negative or equivocal MRI has different implications at low versus high PSAD [8,9].

Percent free PSA similarly provides additional discrimination in men with moderately elevated total PSA. The classic multicenter study demonstrated that a 25% free-PSA threshold retained 95% sensitivity while improving specificity in men with PSA 4–10 ng/mL [24]. However, this threshold was developed in a specific historical population and should now be interpreted alongside newer risk calculators, MRI, and other available biomarkers.

#### 4.1.2. Adjunctive Blood- and Urine-Based Biomarkers

Secondary biomarkers are most useful when the probability of GG ≥ 2 disease is sufficiently uncertain that an additional test could reasonably alter the decision to biopsy. They are less useful when baseline risk is already sufficiently low to justify surveillance or sufficiently high that biopsy is indicated regardless of the biomarker result. AUA/SUO guidance estimates that, across the biomarker evidence base, using secondary markers can reduce biopsies by approximately one third but is accompanied by delayed or missed detection of a small proportion of clinically significant cancers [9]. Thus, biopsy reduction and missed-cancer risk should be considered together rather than reporting NPV or sensitivity in isolation.

Among blood-based assays, PHI combines total PSA, free PSA, and [-2]proPSA, while the 4Kscore integrates total, free, and intact PSA, human kallikrein 2, and clinical variables. Both have demonstrated better discrimination for higher-grade cancer than total PSA alone [25,26]. IsoPSA evaluates structural isoforms of PSA and has demonstrated prospective multicenter performance for GG ≥ 2 detection [27]. These tests should not be considered interchangeable based on AUC alone because study populations, analytical platforms, thresholds, and clinical pathways differ.

MiCheck incorporates PSA, percent free PSA, digital rectal examination, and human epididymis protein 4 (HE4). In the published MiCheck-01 cohort, the assay achieved an AUC of 0.85 for clinically significant prostate cancer [28]; however, its external-validation evidence and clinical adoption remain less mature than those of more established adjunctive biomarkers.

Urine-based assays provide a different molecular approach. SelectMDx incorporates urinary HOXC6 and DLX1 expression with clinical variables, whereas ExoDx Prostate IntelliScore measures urinary exosomal PCA3, ERG, and SPDEF transcripts without requiring a preceding DRE [29,30]. Importantly, modern studies evaluating these assays alongside MRI demonstrate that the incremental value of a biomarker depends on the surrounding diagnostic pathway. In the prospective SelectMDx study, an MRI-only strategy produced greater biopsy avoidance and missed fewer GG ≥ 2 cancers than SelectMDx alone, illustrating why biomarker performance derived from a pre-MRI diagnostic era cannot automatically be extrapolated to an MRI-integrated pathway [29].

Accordingly, no individual blood or urine biomarker should be described as universally superior. Test selection should reflect the clinical question, prior biopsy status, MRI availability, local validation, cost, and whether the result would materially change management.

#### 4.1.3. Multiparametric MRI and PI-RADS

Prostate MRI has become central to contemporary evaluation of men in whom biopsy is being considered. Multiparametric MRI combines high-resolution T2-weighted imaging with diffusion-weighted imaging and apparent diffusion coefficient mapping; dynamic contrast enhancement provides additional information in conventional mpMRI protocols [32]. Interpretation is standardized using PI-RADS, currently version 2.1 [33].

A major strength of PI-RADS is that it communicates graded probability rather than a binary imaging diagnosis. Pooled evidence summarized by the AUA/SUO demonstrates a progressive increase in GG ≥ 2 prevalence from approximately 7% for PI-RADS 1–2 to 70% for PI-RADS 5 [9]. Thus, an MRI report should not simply be categorized as “positive” or “negative” without considering PI-RADS category and underlying clinical risk.

Conversely, a negative MRI does not exclude significant cancer. In biopsy-naïve men, the pooled NPV of PI-RADS 1–2 for GG ≥ 2 disease is approximately 91%, meaning that roughly one in ten men with a negative MRI may nevertheless harbor GG ≥ 2 cancer, with substantial variation according to individual risk [9]. A negative MRI can therefore support biopsy avoidance in appropriately selected lower-risk patients, but men with persistently elevated clinical risk, including elevated PSAD, strong family history, germline susceptibility, abnormal examination, or other concerning findings, may still warrant systematic biopsy.

MRI performance is also influenced by reader expertise, scanner quality, acquisition technique, lesion location, prostate size, and tumor phenotype. Small-volume tumors, infiltrative lesions, and some transition-zone cancers may be less conspicuous. Histologic variants discussed in Section 2 may also generate discordance between PSA, conventional MRI appearance, and tumor burden. These limitations reinforce the need to interpret MRI as one component of integrated risk assessment rather than as an infallible reference standard.

#### 4.1.4. MRI-Directed and Systematic Biopsy

Histopathological examination remains necessary for definitive diagnosis in most patients with suspected primary prostate cancer. For men with MRI-visible lesions, targeted biopsy increases the yield of clinically significant disease while reducing detection of GG1 tumors compared with systematic biopsy alone. In the PRECISION randomized trial, the MRI pathway detected clinically significant cancer in 38% of participants compared with 26% in the standard-biopsy group, while clinically insignificant cancer was detected substantially less frequently [34].

Targeted biopsy and systematic biopsy should nevertheless not be viewed as mutually exclusive techniques. Targeted cores preferentially characterize MRI-visible lesions, whereas systematic sampling can identify clinically significant MRI-occult tumors and can reduce histologic undergrading caused by targeting or registration error. Current AUA/SUO guidance recommends targeted biopsy of suspicious MRI lesions in biopsy-naïve men and allows the addition of systematic template sampling according to the clinical setting [9].

The route of biopsy has also evolved. Transrectal biopsy historically dominated US practice but carries an inherent infectious risk because the needle traverses rectal mucosa. The transperineal approach avoids rectal traversal and can now be performed under local anesthesia in outpatient practice. In PREVENT, transperineal biopsy without prophylactic antibiotics produced no infections, while clinically significant cancer detection was comparable with transrectal biopsy performed using targeted antimicrobial prophylaxis [35]. These findings support wider adoption of transperineal biopsy, particularly in settings prioritizing antimicrobial stewardship, although both routes remain technically acceptable depending on expertise and clinical circumstances.

#### 4.1.5. Evaluation After a Previous Negative Biopsy

A previous negative biopsy decreases but does not eliminate the probability of clinically significant prostate cancer. PSA alone should not be used as the sole trigger for repeat biopsy [9]. Re-evaluation should incorporate the protective effect of the prior negative biopsy together with current PSA and PSAD, MRI findings, age, family history, pathological findings from the previous biopsy, and other relevant risk factors.

For men who have not previously undergone MRI, mpMRI is particularly useful before repeat biopsy because it can identify lesions missed by systematic sampling and enable targeted tissue acquisition. When MRI remains negative but clinical suspicion is sufficiently high, systematic repeat biopsy may still be appropriate [9].

Tissue-based epigenetic testing has also been evaluated in this setting. ConfirmMDx measures methylation of GSTP1, APC, and RASSF1 in histologically benign biopsy cores to detect a molecular “field effect” associated with nearby occult carcinoma [31]. The MATLOC study reported an NPV of 90% for cancer on repeat biopsy. This result should be interpreted carefully: it was generated in an older retrospective cohort and the endpoint was repeat-biopsy cancer rather than specifically contemporary GG ≥ 2 disease. ConfirmMDx is therefore best positioned as a selective adjunct after a negative biopsy, not as a general screening test.

Pathologic findings such as atypical small acinar proliferation, atypical intraductal proliferation, intraductal carcinoma, or other suspicious lesions may independently alter the need and timing of further diagnostic evaluation. In particular, the presence of IDC-P or concerning cribriform/intraductal morphology should not be treated as equivalent to a routine benign biopsy [10,19,20].

### 4.2. Post-Biopsy Characterization and Risk Stratification

#### 4.2.1. Histopathology and Integrated Clinical Risk

Once prostate cancer is confirmed, histopathological interpretation becomes the foundation of disease characterization. The ISUP Grade Group system translates Gleason architecture into five prognostically distinct categories: GG1 corresponds to Gleason 3 + 3; GG2 to 3 + 4; GG3 to 4 + 3; GG4 predominantly to Gleason 8; and GG5 to Gleason 9–10 [36]. Importantly, GG1 should not simply be described as “indolent,” because biological behavior also depends on tumor volume, stage, PSA characteristics, MRI findings, sampling adequacy, and other pathological features.

Grade Group should therefore be integrated with PSA, clinical T stage, extent of biopsy involvement, imaging findings, and other relevant factors. Clinical risk models such as CAPRA provide reproducible multivariable estimates and can improve prognostic communication beyond any individual variable [37].

Morphology provides additional information not fully captured by Grade Group alone. IDC-P and invasive cribriform architecture are associated with adverse biology and should be specifically recognized and reported [11,19,20]. Similarly, ductal or neuroendocrine differentiation may generate clinically important discordance between PSA, imaging, morphology, and disease burden [10,11,12]. This is one area in which an integrated precision-diagnostic approach provides greater depth than a conventional PSA–Gleason–stage framework.

#### 4.2.2. Tissue-Based Genomic Classifiers

Tissue-based genomic classifiers should be distinguished from diagnostic biomarkers used to decide whether a biopsy is necessary. Decipher, Oncotype DX Genomic Prostate Score, and Prolaris are principally prognostic assays used after prostate cancer has already been identified. Their role is to provide biological information that may refine risk when conventional clinicopathological factors leave meaningful uncertainty.

Decipher derives a genomic risk score from expression of 22 genes associated with several pathways involved in tumor progression. In biopsy tissue, the score has demonstrated independent association with subsequent metastatic risk, although early biopsy studies involved relatively selected cohorts [38]. The Genomic Prostate Score uses a 17-gene expression panel spanning several biological pathways and has demonstrated associations with adverse surgical pathology independent of conventional clinical variables [39]. Prolaris evaluates cell-cycle progression genes; independent biopsy validation has shown an association between increasing CCP score and prostate cancer-specific mortality after adjustment for clinical risk [40].

These assays should not be presented as universally indicated or as replacements for Grade Group, MRI, PSA, or clinical risk assessment. Their greatest potential utility is in selected patients in whom the genomic result could meaningfully alter the estimated probability of progression or influence shared decision-making. Cost, tissue requirements, assay-specific endpoints, tumor heterogeneity, and differences between validation populations also limit direct comparison of scores across commercial platforms.

#### 4.2.3. PSMA PET/CT for Initial Staging

PSMA PET/CT has substantially changed molecular staging of prostate cancer but should be positioned according to clinical indication. It is not a screening test and does not replace histological confirmation of a primary prostate lesion. Its strongest established diagnostic roles are staging selected patients with higher-risk biopsy-confirmed disease and localizing disease in biochemical recurrence.

The randomized proPSMA trial provides high-level evidence in men with high-risk prostate cancer being considered for curative-intent treatment. ^68^Ga-PSMA-11 PET/CT demonstrated an overall staging accuracy of 92% compared with 65% for conventional CT and bone scintigraphy, with higher sensitivity and specificity for pelvic nodal or distant metastatic disease [13]. The study therefore established the superiority of PSMA PET/CT over conventional staging imaging in this selected population.

Nevertheless, a negative PSMA PET does not exclude microscopic nodal metastasis, and sensitivity depends on lesion size and biological PSMA expression. Poorly differentiated and neuroendocrine phenotypes may show reduced or heterogeneous PSMA expression [12]. These limitations are particularly relevant when pathology, PSA, anatomic imaging, and PSMA PET findings are discordant.

### 4.3. Disease Monitoring, Recurrence, and Advanced-Disease Diagnostics

#### 4.3.1. Active Surveillance

Active surveillance is a guideline-supported and generally preferred management strategy for appropriately selected men with low-risk prostate cancer, but it should not be described as implying that all GG1 tumors are biologically harmless or that immediate treatment never provides benefit [7,8,9]. The objective is to defer or avoid treatment-related morbidity while maintaining the opportunity for curative intervention if biological or pathological progression becomes evident.

Contemporary active surveillance therefore relies on longitudinal reassessment rather than passive observation. Components may include serial PSA measurements, clinical review, repeat MRI, and confirmatory or surveillance biopsy; exact intervals vary according to guideline, institutional protocol, patient age, baseline risk, MRI findings, and previous biopsy results.

Recent Canary PASS data provide strong prospective support for this approach. Among 2155 men managed with protocol-directed active surveillance, the 10-year cumulative incidence of metastasis was 1.4% and prostate cancer-specific mortality was 0.1%, while approximately 43% experienced biopsy grade reclassification and 49% ultimately underwent treatment [41]. These findings demonstrate both the safety of structured surveillance for favorable-risk disease and the importance of continued monitoring.

Long-term randomized evidence from ProtecT provides complementary context but should not be equated directly with contemporary protocol-driven active surveillance. Fifteen-year prostate cancer mortality was low across active monitoring, surgery, and radiotherapy groups, while metastatic progression and clinical progression occurred more frequently in the active-monitoring group [42]. Thus, the evidence supports careful risk-adapted surveillance rather than the categorical assertion that definitive treatment confers no clinically meaningful benefit.

#### 4.3.2. Biochemical Recurrence and Molecular Restaging

Following definitive local therapy, serum PSA remains the principal biomarker for detecting biochemical recurrence. After radical prostatectomy, contemporary AUA/ASTRO/SUO guidance defines biochemical recurrence as a PSA of at least 0.2 ng/mL with a confirmatory value above 0.2 ng/mL, while recognizing that ultrasensitive assays may identify lower PSA concentrations before the conventional definition is met [43]. The distinction between a detectable ultrasensitive PSA and formally defined biochemical recurrence is important because it prevents overinterpretation of very low-level PSA signals.

After radiotherapy, biochemical failure is conventionally defined by the Phoenix criterion as a PSA increase of at least 2 ng/mL above the post-treatment nadir [44]. PSA kinetics, including PSA doubling time, provide additional prognostic information and should be interpreted alongside the original Grade Group, pathological stage, time to recurrence, prior treatment, and current imaging.

PSMA PET/CT has become particularly important for localization of biochemical recurrence [14]. However, the probability of identifying disease is strongly dependent on PSA concentration and other clinical factors. PSMA PET can detect disease at PSA concentrations below 0.5 ng/mL, but detection is substantially less frequent than at higher PSA levels. Therefore, low-PSA detection should be described as possible and clinically useful in selected patients, rather than implying uniformly high sensitivity at a specific minimal PSA threshold.

PSMA PET findings can identify local recurrence, pelvic nodal disease, or distant metastases that were occult on conventional imaging, but imaging localization and therapeutic benefit are distinct evidentiary questions. The detection of a lesion should not, by itself, be interpreted as proof that PSMA PET-directed intervention improves long-term survival.

#### 4.3.3. Molecular Characterization of Advanced and Metastatic Disease

Advanced prostate cancer requires a broader diagnostic framework than localized disease because tumor evolution can generate actionable genomic alterations, treatment-resistance phenotypes, and lineage transformation. Current evidence supports both germline and somatic genomic testing in metastatic prostate cancer, using appropriately validated multigene panels [45]. Germline testing also has implications beyond the affected patient through hereditary cancer assessment and cascade testing of relatives.

Somatic testing may be performed using tumor tissue or, when adequate tissue is unavailable or unsuitable, circulating tumor DNA from plasma can provide a complementary molecular source [45]. Clinically relevant alterations include homologous recombination repair genes such as BRCA1, BRCA2, ATM, PALB2, and others, as well as mismatch repair deficiency or microsatellite instability. However, the clinical significance of an alteration is gene-, assay-, disease-, and indication-specific. A mutation in an HRR-associated gene should therefore not be described generically as automatically “qualifying” a patient for a PARP inhibitor; eligibility depends on the specific therapeutic indication and applicable regulatory criteria.

Liquid biopsy has particular value in advanced disease because greater tumor burden and systemic shedding increase the likelihood that ctDNA will adequately represent tumor genomic alterations. In localized prostate cancer, by contrast, low ctDNA concentrations continue to limit sensitivity, and liquid biopsy should remain categorized as an emerging rather than established early-detection strategy. Plasma ctDNA can complement tissue genomic profiling when adequate tumor tissue is unavailable or unsuitable; however, a negative plasma result in the setting of a low tumor fraction does not exclude a clinically relevant alteration [45]. 

Finally, unexpected clinical behavior should prompt consideration of phenotypic re-evaluation. Rapid progression, visceral metastases, bulky disease disproportionate to PSA, or loss of conventional androgen-receptor/PSMA features may raise concern for neuroendocrine or other treatment-emergent lineage transformation. In such cases, repeat tissue biopsy and appropriate histopathological and immunohistochemical characterization can provide information that genomic testing or serum PSA alone cannot supply [12].

## 5. Clinical Application of Artificial Intelligence and Emerging Diagnostic Modalities

The diagnostic technologies discussed in this section should be distinguished from the established components of the prostate cancer pathway described in Section 4. Artificial intelligence (AI), digital pathology, liquid biopsy, micro-ultrasound, alternative molecular radiotracers, and multi-omic approaches have varying levels of clinical validation and adoption. Some have undergone substantial external or prospective validation, whereas others remain investigational. Their clinical value should therefore be considered according to the intended use, quality of validation, regulatory status, reproducibility across institutions, and evidence that their use improves diagnostic decision-making.

### 5.1. Artificial Intelligence in Imaging and Pathology

#### 5.1.1. AI-Assisted Interpretation of Prostate MRI

Interpretation of prostate MRI remains dependent on image quality and radiologist expertise, and interobserver variability persists despite standardization through PI-RADS [33]. AI-based systems have therefore been developed to assist with prostate segmentation, lesion detection, lesion characterization, and estimation of the probability of clinically significant prostate cancer (csPCa).

The strongest evidence for AI-based prostate MRI currently comes from large multicenter validation studies rather than from small single-center algorithm-development datasets. In the international PI-CAI study, an AI system achieved an area under the receiver-operating-characteristic curve (AUROC) of approximately 0.91 for detection of GG ≥ 2 prostate cancer, compared with approximately 0.86 for radiologists participating in the reader study [15]. These findings demonstrate that AI can achieve high diagnostic discrimination under controlled validation conditions but do not establish autonomous AI interpretation as a replacement for radiologists.

Regulatory status must also be considered at the level of the individual software product. For example, AI-Rad Companion Prostate MR has received US Food and Drug Administration (FDA) 510(k) clearance as radiological image-processing software intended to support processing, analysis, and annotation of prostate MR images [46,47]. Such clearance applies to the specific device and intended use and should not be interpreted as establishing AI-assisted prostate MRI as a universal standard-of-care diagnostic pathway.

Reader-assistance studies suggest that the principal near-term role of AI may be as a second reader or decision-support tool. In a two-center study involving 900 patients and radiologists with different levels of prostate MRI experience, AI assistance increased lesion-level sensitivity among less-experienced readers from 0.78 to 0.88 and patient-level AUC from 0.84 to 0.89. Median interpretation time decreased from 250 to 130 s [48]. The benefit was less pronounced among experienced prostate MRI readers, suggesting that AI may have particular value in reducing experience-dependent variability rather than uniformly improving all readers.

Generalizability across institutions, scanner platforms, acquisition protocols, and patient populations remains an important limitation of prostate MRI AI. These implementation, bias, and post-deployment monitoring considerations are discussed in Section 6.3.

#### 5.1.2. Digital Pathology and AI-Assisted Histopathologic Assessment

Digitization of histopathology through whole-slide imaging (WSI) has enabled development of AI algorithms for prostate cancer detection, tumor localization, Gleason-pattern recognition, and Grade Group assignment. These applications are particularly attractive because conventional prostate biopsy interpretation can be affected by interobserver variability, especially in distinguishing adjacent Gleason patterns and evaluating small foci of carcinoma.

External validation studies have demonstrated encouraging performance. In an international multi-institutional study involving 5922 hematoxylin-and-eosin sections representing 7473 prostate biopsy cores, an AI classifier achieved sensitivities ranging from 0.971 to 1.000 and specificities from 0.875 to 0.976 for tumor detection across external cohorts [49]. Agreement for Gleason grading was within the range observed among experienced genitourinary pathologists, although discordant cases remained and performance was influenced by artifacts and difficult cancer mimics [49].

Independent validation of another prostate biopsy AI system similarly demonstrated potential clinical utility. In 593 whole-slide biopsy images, AI-assisted interpretation improved the agreement of a general pathologist with expert reference Grade Groups and reduced slide-review time [50]. These findings support a potential role for AI in screening slides, highlighting suspicious areas, providing a second assessment of grade, and improving workflow efficiency.

However, automated pathology should not be equated with autonomous diagnosis. Histologic interpretation extends beyond cancer detection and Gleason grading to recognition of intraductal carcinoma, cribriform architecture, treatment effects, unusual variants, neuroendocrine differentiation, inflammatory mimics, and other features that may require morphologic context, immunohistochemistry, and clinical correlation. Scanner type, staining variability, tissue preparation, artifacts, and domain shift can also alter algorithm performance. Accordingly, AI-based pathology is best regarded as an emerging decision-support and quality-assurance technology operating under pathologist oversight.

### 5.2. Next-Generation and Emerging Diagnostic Modalities

#### 5.2.1. Liquid Biopsy for Molecular Profiling and Disease Monitoring

Liquid biopsy encompasses analysis of circulating tumor DNA (ctDNA), cell-free DNA (cfDNA), circulating tumor cells (CTCs), extracellular vesicles, and other tumor-derived circulating material. Its potential advantage is the ability to characterize molecular disease noninvasively and repeatedly, potentially capturing genomic heterogeneity that may not be represented by a single tissue biopsy.

The clinical relevance of liquid biopsy differs substantially between localized and advanced prostate cancer. In localized disease, the fraction of tumor-derived DNA in plasma is often very low because of limited systemic tumor shedding. Consequently, ctDNA-based approaches for initial cancer detection, discrimination of aggressive localized disease, minimal residual disease assessment, and active-surveillance monitoring remain investigational [51]. Research strategies incorporating methylation, fragmentomics, copy-number alterations, and mutation profiles are promising, but analytical sensitivity, preanalytical standardization, prospective validation, and clinically actionable thresholds remain unresolved.

In metastatic disease, particularly metastatic castration-resistant prostate cancer (mCRPC), ctDNA is more readily detectable and can provide clinically useful somatic genomic information. Contemporary guidance supports somatic genomic testing in metastatic prostate cancer, with plasma ctDNA representing a complementary option when adequate tumor tissue is unavailable or difficult to obtain [45]. However, a negative plasma result in a patient with low ctDNA fraction does not reliably exclude a tumor alteration and may require tissue-based testing.

CTC-based analysis provides another approach to molecular characterization. AR-V7 is a truncated androgen-receptor splice variant lacking the conventional ligand-binding domain. Prospective multicenter evidence has shown that CTC-detected AR-V7 is associated with reduced benefit from androgen-receptor signaling inhibitors and with adverse clinical outcomes in mCRPC [46]. This association should not, however, be interpreted as an automatic treatment-selection rule. AR-V7 is not a universal stand-alone companion diagnostic directing patients to taxanes, PARP inhibitors, or PSMA-targeted radiopharmaceutical therapy. Treatment selection requires integration of prior therapy, genomic findings, disease phenotype, PSMA expression, and agent-specific regulatory indications.

Thus, liquid biopsy currently has its strongest role in molecular characterization of advanced disease, whereas its use for early detection and localized-disease surveillance remains an emerging research application.

#### 5.2.2. High-Frequency Micro-Ultrasound and PRI-MUS

High-frequency micro-ultrasound (micro-US) provides real-time prostate imaging at 29 MHz and permits lesion assessment and targeted biopsy during the same procedure. Unlike conventional ultrasound, which is primarily used to guide needle placement, micro-US is intended to visualize tissue features associated with prostate cancer directly.

The Prostate Risk Identification using Micro-Ultrasound (PRI-MUS) system was developed to standardize interpretation of micro-US findings. The original PRI-MUS protocol assigns increasing levels of suspicion according to sonographic tissue characteristics, with higher scores corresponding to a greater probability of clinically significant cancer [52]. As with PI-RADS, however, the score should be interpreted within the clinical context rather than treated as an independent histological diagnosis.

The strongest comparative evidence is provided by the OPTIMUM randomized clinical trial. In biopsy-naïve men, micro-US-guided biopsy was noninferior to MRI-fusion-guided biopsy for detection of GG ≥ 2 prostate cancer, with clinically significant cancer detected in approximately 46% and 43% of participants, respectively [16]. This trial substantially strengthens the evidence base for micro-US compared with earlier single-center and observational studies.

More recently, a secondary analysis of the OPTIMUM trial provided external validation of the PRI-MUS classification in 347 patients and 4590 biopsy cores [53]. Using PRI-MUS 1–2 versus 3–5, per-core sensitivity, specificity, positive predictive value, and negative predictive value for csPCa were 72%, 74%, 37%, and 93%, respectively, with an AUC of 0.78. Importantly, these values are per-core estimates from this specific analysis and should not be generalized as universal patient-level diagnostic performance.

Micro-US therefore represents a credible emerging alternative or adjunct to MRI-based biopsy pathways, particularly where MRI is unavailable, delayed, contraindicated, or difficult to access. Nevertheless, MRI remains more extensively integrated into contemporary guidelines and has a broader evidence base for whole-gland assessment and staging. Operator training, interpretation standards, anterior and transition-zone assessment, equipment availability, and reproducibility across practice settings remain important considerations before micro-US can be regarded as interchangeable with MRI in all diagnostic settings.

#### 5.2.3. Emerging Molecular Radiotracers and Imaging of PSMA-Low Disease

PSMA PET/CT has an established role in selected staging and biochemical-recurrence settings, as discussed in Section 4 [13,14]. Emerging molecular imaging research is therefore increasingly focused on improving tracer characteristics and identifying biological targets that may provide useful information in tumors with limited or heterogeneous PSMA expression.

Fibroblast activation protein (FAP) is expressed by activated fibroblasts within the tumor microenvironment and has become a target for PET radiotracers based on fibroblast activation protein inhibitors (FAPIs). Published reports have described FAPI PET uptake in selected genitourinary malignancies, including small numbers of patients with prostate cancer [54]. However, prostate-specific evidence remains preliminary, and FAPI PET should currently be regarded as investigational rather than an established alternative to PSMA PET.

Alternative metabolic imaging may also be relevant in selected aggressive phenotypes. Neuroendocrine and treatment-emergent lineage-transformed prostate cancers can demonstrate reduced or heterogeneous PSMA expression, limiting the reliability of PSMA-targeted imaging in some patients [12]. ^18^F-fluorodeoxyglucose (^18^F-FDG) PET can provide complementary information in highly glycolytic, poorly differentiated, or neuroendocrine disease, while radiolabeled somatostatin-receptor ligands may be informative in selected neuroendocrine tumors [55]. The appropriate tracer depends on the pathological phenotype and clinical scenario; no alternative PET tracer should be presented as universally superior for PSMA-low disease.

These approaches illustrate an important principle of precision imaging: molecular imaging performance depends on tumor phenotype and target expression. Discordance among PSA, histology, PSMA PET, and clinical progression should therefore prompt reconsideration of tumor biology rather than simple substitution of one imaging modality for another.

#### 5.2.4. Multi-Omic Integration and Precision Diagnostic Models

Multi-omic approaches integrate information from two or more molecular layers, including geonomics, transcriptomics, epigenomics, proteomics, metabolomics, and, increasingly, spatial or single-cell datasets. In prostate cancer, these approaches have improved understanding of molecular heterogeneity and have generated candidate biomarkers and composite signatures for disease detection, prognostic classification, and biological characterization [56].

The potential diagnostic advantage of multi-omic integration is that a single molecular marker may incompletely represent a heterogeneous tumor. Combining genomic alterations with RNA-expression patterns, protein abundance, epigenetic changes, metabolic signatures, imaging features, and clinicopathological variables may provide a more comprehensive representation of disease biology than any individual data type.

However, most multi-omic prostate cancer models remain at the discovery or translational-validation stage. Major barriers include small or highly selected cohorts, high-dimensional datasets, batch effects, platform heterogeneity, incomplete external validation, computational complexity, lack of standardized analytical pipelines, and uncertain incremental clinical utility over established clinical, imaging, and genomic models [56]. AI and machine-learning methods can facilitate integration of these complex datasets, but they also increase concerns regarding overfitting, interpretability, reproducibility, and population bias.

Accordingly, multi-omic models should currently be viewed as emerging precision-diagnostic frameworks rather than established clinical tests. Their eventual clinical adoption will require prospective external validation, predefined clinically meaningful endpoints, reproducible analytical pipelines, and demonstration that the integrated information improves decision-making beyond validated conventional approaches.

## 6. Challenges and Future Directions

### 6.1. From Diagnostic Accuracy to Clinical Utility

A central challenge in precision prostate cancer diagnostics is moving from promising test performance to evidence of meaningful clinical utility. High sensitivity, specificity, negative predictive value, or area under the receiver-operating-characteristic curve does not necessarily demonstrate that implementation of a diagnostic test improves patient care. Diagnostic performance is influenced by disease prevalence, patient selection, threshold definition, reference standard, and clinical setting; transparent reporting of these factors is essential for interpreting diagnostic studies [57].

The evidence required for clinical translation should therefore reflect the intended use of the technology. Early development requires analytical validity and reproducibility, followed by evaluation in appropriately defined clinical populations. Diagnostic tests intended to identify GG ≥ 2 disease should undergo prospective or external validation against a suitable reference standard, ideally across independent institutions and populations. Technologies intended to alter clinical decision-making require an additional level of evidence demonstrating that their use meaningfully changes management without producing unacceptable downstream harms.

Relevant clinical-utility endpoints depend on where a technology enters the diagnostic pathway. For a pre-biopsy biomarker, meaningful endpoints may include the proportion of biopsies avoided together with the proportion of GG ≥ 2 cancers delayed or missed. For MRI or image-guided biopsy technologies, clinically relevant measures include GG ≥ 2 detection, detection of clinically insignificant disease, repeat procedures, complications, and diagnostic delay. For genomic classifiers, meaningful evaluation should determine whether the result adds information beyond established clinicopathological models and whether this information changes management. For AI systems, evaluation should consider not only standalone algorithm performance but also the performance of the human–AI clinical workflow.

Randomized controlled trials are valuable when a diagnostic strategy is expected to alter downstream management or patient outcomes, as illustrated by trials such as PRECISION, PREVENT, and OPTIMUM [16,34,35]. However, an RCT is not universally required for every diagnostic device or biomarker. Depending on the clinical question, well-designed prospective diagnostic-accuracy studies, multicenter external-validation studies, comparative-effectiveness studies, pragmatic trials, or prospective implementation studies may provide the appropriate level of evidence. Early live evaluation of AI clinical decision-support systems similarly requires assessment of safety, workflow integration, human factors, and clinical performance before progression to larger-scale studies [58].

Reporting standards are equally important. Diagnostic-accuracy studies should adhere to established frameworks such as STARD [57], whereas studies developing or evaluating clinical prediction models, including machine-learning models, should follow contemporary reporting guidance such as TRIPOD + AI [59]. These frameworks improve transparency but should not be confused with proof of clinical utility: complete reporting facilitates critical appraisal, whereas clinical utility must be demonstrated through evidence appropriate to the intended use.

Longitudinal validation is particularly important in prostate cancer because clinically relevant outcomes may emerge years after the initial diagnostic decision. Avoidance of an immediate biopsy is beneficial only if clinically significant disease is not systematically delayed, and reclassification of an individual to a lower-risk category is valuable only if it does not compromise subsequent cancer control. Future studies should therefore connect short-term diagnostic endpoints with longer-term outcomes such as repeat biopsy, grade reclassification, metastatic progression, treatment burden, patient-reported outcomes, and prostate cancer-specific outcomes where feasible.

### 6.2. Healthcare Equity, Access, and Generalizability

Precision diagnostics may improve risk stratification while simultaneously creating new access disparities if advanced technologies are concentrated among patients and healthcare systems with greater resources. Existing racial, geographic, socioeconomic, and healthcare-access inequities in prostate cancer have been well documented [22]. The introduction of costly or infrastructure-dependent technologies therefore requires assessment not only of diagnostic performance but also of who can realistically access them.

MRI illustrates this challenge. High-quality prostate MRI depends on appropriate equipment, standardized acquisition, and experienced interpretation. PSMA PET/CT additionally requires specialized radiopharmaceutical and nuclear-medicine infrastructure. Tissue genomic classifiers and commercial molecular assays may be influenced by insurance coverage, reimbursement policies, laboratory availability, and out-of-pocket costs. Even when a diagnostic technology demonstrates favorable accuracy, limited accessibility may restrict its real-world clinical impact.

Emerging alternatives should not automatically be assumed to improve equity simply because they appear less infrastructure-intensive. For example, micro-ultrasound can provide real-time lesion visualization and biopsy guidance without requiring a separate MRI examination and has now demonstrated randomized comparative evidence [16]. Whether this translates into improved accessibility, lower total diagnostic costs, or narrower geographic disparities, however, requires formal health-economic and implementation evaluation rather than assumption.

Equitable validation is equally important. Diagnostic tests and AI systems should be evaluated in populations reflecting the diversity of patients in whom they will ultimately be used. Differences in ancestry, age, prostate volume, disease prevalence, comorbidity, imaging equipment, pathology practice, and healthcare setting may all influence performance. A test evaluated primarily in highly selected tertiary-center populations may therefore perform differently when introduced into community practice.

Future implementation studies should incorporate measures of diagnostic access and equity as predefined outcomes, including time from abnormal PSA to definitive diagnosis, access to MRI or biopsy, geographic availability, insurance-related barriers, diagnostic completion rates, and performance across relevant demographic groups. Equity should therefore be considered part of external validity and clinical implementation rather than an issue addressed only after adoption.

### 6.3. AI Validation, Algorithmic Bias, Transparency, and Regulatory Oversight

AI presents additional challenges because model performance depends on the data-generating environment in which the algorithm was developed and evaluated. Prostate MRI algorithms may be sensitive to scanner manufacturer, magnetic-field strength, acquisition protocol, reconstruction method, disease prevalence, annotation strategy, and radiologist expertise. Digital-pathology algorithms may similarly be affected by fixation, staining, scanner characteristics, tissue preparation, and institutional pathology practices [15,48,49,50].

A major methodological concern is dataset shift, in which the population or technical environment encountered after deployment differs from the development dataset. High performance in an internal retrospective test set therefore does not establish generalizability. Independent external evaluation across institutions, equipment platforms, clinical environments, and patient populations is necessary before widespread implementation.

Bias may arise when development datasets inadequately represent important patient groups or clinical settings. Consequently, performance should be reported across clinically relevant subgroups when sample size permits rather than only as an overall AUC. Differences in false-positive and false-negative rates are particularly important because unequal error distributions may translate into differential biopsy rates, delayed diagnoses, or unnecessary procedures.

Contemporary regulatory principles increasingly emphasize a total-product-life-cycle approach to machine-learning-enabled medical devices. FDA and international Good Machine Learning Practice principles recommend representative datasets, appropriate separation of training and test data, fit-for-purpose reference standards, evaluation of the human–AI team, clear information for users, and monitoring of deployed models for changes in performance [60]. These considerations are particularly important for adaptive or updated algorithms whose operating characteristics may evolve after initial deployment.

Transparency is also essential but should not be reduced to a requirement that every complex model provide a simple mechanistic explanation for each prediction. Clinicians should instead understand the intended population, input data, output, operating threshold, known failure modes, validation environment, and appropriate role of the software within the clinical workflow. TRIPOD + AI similarly emphasizes transparent reporting of development and evaluation methods for machine-learning and regression-based prediction models [59].

Human oversight remains necessary. The most clinically plausible near-term applications of prostate AI are therefore likely to be augmentation rather than autonomous replacement: prioritizing suspicious MRI examinations, highlighting candidate lesions, assisting segmentation, supporting Grade Group assessment, or providing quality-control alerts. Prospective studies should evaluate whether such systems improve the performance and efficiency of the combined human–AI team rather than demonstrating only that an algorithm performs well in isolation.

### 6.4. Future Directions: Multimodal Integration, and Theranostic Selection

The next phase of precision prostate cancer diagnostics is likely to involve integration rather than continued development of isolated tests. Clinical risk variables, longitudinal PSA data, MRI features, biopsy morphology, tissue genomics, circulating biomarkers, and molecular imaging provide complementary information reflecting different aspects of tumor biology. The future challenge is determining whether combining these data materially improves clinical decision-making compared with simpler validated approaches.

Future diagnostic models may combine clinical risk variables, imaging, pathology, and molecular data. The value of such multimodal approaches should be determined by their incremental performance and clinical utility relative to simpler validated models rather than by model complexity alone [56].

Molecular imaging also illustrates the convergence of diagnosis and treatment selection through theranostics. PSMA PET can characterize the distribution and intensity of PSMA-expressing disease and, in selected advanced-disease settings, can identify patients considered for PSMA-targeted radioligand therapy. The VISION trial exemplified this diagnostic–therapeutic linkage by requiring PSMA-positive metastatic castration-resistant prostate cancer on ^68^Ga-PSMA-11 PET/CT before treatment with ^177^Lu-PSMA-617 [61]. The trial demonstrated improved radiographic progression-free and overall survival when ^177^Lu-PSMA-617 was added to protocol-permitted standard care, establishing a clinically important role for target-expression imaging in treatment selection [61].

The diagnostic lesson extends beyond PSMA. Precision imaging increasingly seeks to characterize biological phenotype rather than anatomy alone. PSMA expression, metabolic activity, stromal activation, receptor expression, and other molecular characteristics may eventually guide selection among diagnostic and therapeutic strategies. Nevertheless, target visualization should not automatically be equated with therapeutic benefit; imaging biomarkers require indication-specific validation linking target expression to clinically meaningful outcomes.

Ultimately, the future of prostate cancer diagnostics is likely to depend less on identifying a single superior biomarker and more on developing clinically interpretable, validated, and accessible combinations of complementary tests. Successful translation will require rigorous comparative evaluation, external validation, regulatory oversight, economic assessment, equitable implementation, and evidence that added diagnostic complexity produces measurable benefit for patients.

## 7. Conclusions

Prostate cancer diagnosis now relies on a risk-adapted pathway integrating PSA-based assessment, prostate MRI, image-guided biopsy, histopathology and, when clinically indicated, molecular imaging and selected genomic tools. These modalities should be applied according to the specific clinical question rather than as a uniform sequence of tests.

Secondary biomarkers and genomic classifiers may refine decision-making in selected patients, whereas AI-assisted imaging, digital pathology, micro-ultrasound, liquid biopsy for localized disease, alternative molecular radiotracers, and multi-omic models remain at different stages of validation and adoption. Integrated assessment is particularly important when PSA, imaging, histopathology, and clinical behavior are discordant.

Further adoption of emerging diagnostic technologies should depend on external validation, demonstrated incremental clinical utility, regulatory clarity, cost-effectiveness, and equitable access. New tests should ultimately show that they improve risk classification or clinical decision-making beyond established approaches.

## Figures and Tables

**Table 1:** Evidence-based positioning of major diagnostic modalities across the prostate cancer diagnostic pathway.

Diagnostic Modality	Intended Population/Clinical Setting	Representative Cutoff or Decision Point	Diagnostic/Prognostic Endpoint	Representative Study-Specific Performance	Major Limitations/Interpretation	Current Clinical Position in the United States	Key Supporting Evidence
Total PSA	Men undergoing risk-adapted early detection or evaluation for suspected prostate cancer	No universal biopsy cutoff; PSA should be interpreted as a continuous risk marker and a newly elevated value should generally be confirmed before downstream testing	Probability of prostate cancer and GG ≥ 2 disease	A single sensitivity/specificity estimate is not appropriate because performance varies substantially with PSA threshold, age, prostate volume, and population	Limited cancer specificity; affected by BPH, inflammation, urinary retention, instrumentation, and prostate volume	Established initial risk-assessment test	[5,6,7,8,9]
Percent free PSA (%fPSA)	Men with moderately elevated PSA, classically 4–10 ng/mL, particularly with benign DRE	Representative historical cutoff ≤ 25%	Prostate cancer on biopsy	In a prospective multicenter cohort, a 25% cutoff yielded sensitivity 95% (95% CI 92–97%) and specificity 20% (95% CI 16–24%) [24]	Performance depends on PSA range, age, prostate volume, and assay; historical cutoff should not be treated as mandatory	Optional PSA-derived adjunct	[24]
Prostate Health Index (PHI)	Men ≥ 50 years with PSA 4–10 ng/mL and non-suspicious DRE in the validation cohort	PHI 28.6 represented the 90% sensitivity operating point in the cited study	Clinically significant/Gleason ≥ 7 cancer at biopsy	AUC 0.707 for Gleason ≥ 7 disease; at the 90% sensitivity operating point, approximately 30.1% of biopsies for benign or insignificant disease could have been avoided [25]	Threshold-dependent; performance changes with population and MRI incorporation	Guideline-recognized optional pre-biopsy adjunct	[7,8,9,25]
4Kscore	Men referred for prostate biopsy based on clinical suspicion	Continuous probability estimate; biopsy-action threshold should reflect individual risk tolerance rather than a universal cutoff	Gleason ≥ 7/GG ≥ 2 cancer at biopsy	Prospective US multicenter validation: AUC 0.821 (95% CI 0.790–0.852) [26]	Proprietary model; benefit depends on chosen threshold and pretest risk; limited head-to-head data against contemporary MRI-first strategies	Guideline-recognized optional pre-biopsy adjunct	[7,8,9,26]
IsoPSA	Men considered for initial or repeat biopsy	Representative IsoPSA index threshold 6 in prospective validation	GG ≥ 2 cancer	AUC 0.783 (95% CI 0.752–0.814); sensitivity 90.2% (86.4–93.0%); specificity 45.5% (41.4–49.6%); NPV 89.3% (85.6–92.2%) [27]	Performance and clinical utility remain dependent on population prevalence and decision threshold	Guideline-recognized optional adjunct	[7,27]
MiCheck Prostate	Men with elevated PSA being considered for biopsy	Algorithm developed around a 95% sensitivity operating point	Clinically significant cancer, defined as Gleason ≥ 3 + 4	In 358 evaluable MiCheck-01 samples: AUC 0.85, sensitivity 95%, specificity 50% [28]	Development/validation evidence remains less extensive than for established biomarker platforms.	Emerging biomarker	[28]
SelectMDx	Biopsy-naïve men with clinical suspicion of prostate cancer; post-DRE urine	Representative positive risk-score threshold ≥ −2.8 in a prospective multicenter study	GG ≥ 2 cancer	Among 599 biopsy-naïve men, SelectMDx-based biopsy avoidance was 38%, while 10% of GG ≥ 2 cancers would have been missed; in the same study an MRI-only strategy avoided 49% of biopsies while missing 4.9% of GG ≥ 2 cancers [29]	Performance depends on model, threshold, DRE-based collection, and interaction with MRI; incremental benefit may be smaller where high-quality MRI is routinely available	Optional adjunct; not universal standard of care	[7,8,9,29]
ExoDx Prostate IntelliScore (EPI)	Men ≥50 years with PSA 2–10 ng/mL considering initial biopsy; urine collection does not require DRE	Validated cutoff 15.6	GG ≥ 2 cancer	Pooled prospective cohort *n* = 1212: AUC 0.70; NPV 90% at cutoff 15.6; approximately 23% of all biopsies could have been avoided [30]	Predictive values depend on disease prevalence; modest discrimination should be interpreted within multivariable risk assessment	Guideline-recognized optional pre-biopsy adjunct	[7,8,9,30]
ConfirmMDx	Men with persistent suspicion after histologically negative biopsy	Methylation-positive versus methylation-negative assay for GSTP1, APC, and RASSF1	Cancer detected on repeat biopsy	MATLOC cohort *n* = 498: NPV 90% (95% CI 87–93%); assay independently predicted repeat-biopsy outcome, OR 3.17 (95% CI 1.81–5.53) [31]	Original endpoint included cancer on repeat biopsy rather than specifically GG ≥ 2 disease; older retrospective tissue cohorts; not intended for initial biopsy	Selective repeat-biopsy adjunct	[9,31]
mpMRI/PI-RADS	Biopsy-naïve men or men with persistent suspicion after prior negative biopsy	PI-RADS 3–5 generally considered abnormal; local expertise may use PI-RADS 4–5	GG ≥ 2 cancer	Pooled AUA/SUO evidence: GG ≥ 2 prevalence 7% (95% CI 4–11%) for PI-RADS 1–2, 11% (8–14%) for PI-RADS 3, 37% (33–40%) for PI-RADS 4, and 70% (62–79%) for PI-RADS 5; NPV of PI-RADS 1–2 for GG ≥ 2 disease in biopsy-naïve men ≈91% [9]	Reader and center dependence; negative MRI does not exclude significant cancer; lesion conspicuity varies with size, location, histology, and image quality	Central guideline-supported component of contemporary biopsy evaluation	[9,32,33]
MRI-targeted biopsy	Men with suspicious MRI lesion	Targeting generally performed for PI-RADS 3–5 lesions depending on clinical context	GG ≥ 2/clinically significant cancer	PRECISION: csPCa detected in 38% with the MRI pathway versus 26% with standard biopsy; adjusted difference 12 percentage points (95% CI 4–20); clinically insignificant cancer 9% vs. 22% [34]	Targeted biopsy alone can miss or undergrade MRI-occult disease; systematic cores may add information in selected patients	Established for MRI-visible lesions	[9,34]
Transperineal biopsy	Men requiring histological confirmation	Route of tissue sampling rather than diagnostic cutoff	GG ≥ 2 detection and biopsy complications	PREVENT: infection 0% with transperineal biopsy without prophylactic antibiotics vs 1.4% with transrectal biopsy using targeted prophylaxis; difference −1.4% (95% CI −3.2 to 0.3). csPCa detection 53% vs. 50%, adjusted difference 2.0% (95% CI −6 to 10) [35]	Local anesthesia expertise, equipment, procedural learning curve, and transient discomfort	Established biopsy approach; increasingly used because of infectious-safety and antimicrobial-stewardship advantages	[9,35]
ISUP Grade Group	Histologically confirmed prostate cancer	GG1–GG5 based on Gleason patterns	Prognosis and clinicopathologic risk classification	Five-tier Grade Group system demonstrates progressively different recurrence risk and greater clinical interpretability than conventional Gleason categories alone [36]	Sampling error and tumor heterogeneity remain important; architecture such as cribriform growth and IDC-P carries additional information	Core standard pathological classification	[10,19,20,36]
Clinical risk models (e.g., CAPRA)	Histologically confirmed localized disease	Multivariable risk score rather than single biomarker threshold	Recurrence/adverse outcome risk	CAPRA integrates PSA, Gleason/Grade Group, clinical stage, age, and biopsy involvement and provides graded recurrence-risk estimates [37]	Performance depends on population and endpoint; does not capture all molecular or morphologic heterogeneity	Established adjunct to clinicopathologic risk stratification	[37]
Decipher genomic classifier	Selected patients with diagnostic biopsy tissue or prostatectomy tissue when genomic prognosis could change management	Continuous genomic score; risk categories depend on intended clinical use	Metastatic progression	In biopsy tissue, Decipher plus NCCN risk achieved C-index 0.88 (95% CI 0.77–0.96) versus 0.75 (0.64–0.87) for NCCN alone; HR 1.72 (95% CI 1.07–2.81) per 0.1 increase in score [38]	Retrospective validation, selected populations, cost, and tumor sampling; should not replace standard pathology	Selective prognostic adjunct, not routine for every patient	[38]
Oncotype DX Genomic Prostate Score (GPS)	Selected low- to intermediate-risk biopsy-confirmed disease	Continuous 0–100 score; no universal treatment threshold	Adverse pathology/aggressive disease	In validation, OR for high-grade disease 2.3 (95% CI 1.5–3.7) and high-stage disease 1.9 (1.3–3.0) per 20-unit increase in GPS [39]	Prognostic rather than primary diagnostic test; sampling and cost limitations	Selective post-biopsy prognostic adjunct	[39]
Prolaris/cell-cycle progression score	Selected localized biopsy-confirmed disease	Continuous CCP/CCR score	Prostate cancer-specific mortality	Independent needle-biopsy validation: HR 1.76 (95% CI 1.44–2.14) per unit increase in CCP after adjustment for CAPRA [40]	Prognostic, not a screening or primary diagnostic test; clinical impact depends on whether result changes management	Selective post-biopsy prognostic adjunct	[40]
PSMA PET/CT	Selected high-risk initial staging and biochemical recurrence/restaging	No universal PSA cutoff; use depends on clinical indication and pretest probability	Pelvic nodal and distant metastatic disease/recurrent disease localization	proPSMA: accuracy 92% (95% CI 88–95%) vs. 65% (60–69%) for CT plus bone scan; absolute difference 27% (23–31%) [13]. Detection in BCR is PSA-dependent [14]	Limited sensitivity for microscopic disease; heterogeneous/low PSMA expression; not a replacement for biopsy in primary diagnosis	Established molecular imaging for selected staging and recurrence settings	[13,14]
29 MHz micro-ultrasound	Men undergoing prostate biopsy; potential alternative real-time image-guided pathway	PRI-MUS-based lesion assessment	GG ≥ 2 cancer	OPTIMUM: GG ≥ 2 detection 46% with micro-ultrasound-guided biopsy vs. 43% with MRI/conventional ultrasound-guided biopsy; difference 3.52% (95% CI −3.95 to 10.92); met prespecified noninferiority criterion [16]	Operator dependence, training, availability, and less mature guideline integration than MRI	Emerging/alternative image-guided modality with randomized evidence	[16]
AI-assisted prostate MRI	Computer-assisted interpretation of prostate MRI	Algorithm-specific threshold; no universal clinical cutoff	GG ≥ 2 cancer	PI-CAI reader-study subset: AI AUROC 0.91 (95% CI 0.87–0.94) vs. 0.86 (0.83–0.89) for 62 radiologists [15]	Algorithm-, population-, scanner-, and reference-standard dependence; regulatory clearance of individual products does not make AI universally standard of care	Emerging adjunctive technology	[15]

Abbreviations: AUC, area under the receiver-operating-characteristic curve; BPH, benign prostatic hyperplasia; CI, confidence interval; csPCa, clinically significant prostate cancer; DRE, digital rectal examination; GG, Grade Group; IDC-P, intraductal carcinoma of the prostate; MRI, magnetic resonance imaging; NPV, negative predictive value; OR, odds ratio; PHI, Prostate Health Index; PI-RADS, Prostate Imaging Reporting and Data System; PSA, prostate-specific antigen; PSMA PET/CT, prostate-specific membrane antigen positron emission tomography/computed tomography. Performance estimates in this table are study-specific and should not be interpreted as universal characteristics of the corresponding test. Sensitivity, specificity, positive and negative predictive values, biopsy-avoidance rates, and AUC depend on patient selection, disease prevalence, diagnostic threshold, reference standard, MRI quality, and the definition of clinically significant disease. Unless otherwise stated, contemporary studies generally define clinically significant prostate cancer as GG ≥ 2.

## Data Availability

No new data were created or analyzed in this study. Data sharing does not apply to this article.
